# Patients’ Attitudes Toward the Use of Artificial Intelligence as a Diagnostic Tool in Radiology in Saudi Arabia: Cross-Sectional Study

**DOI:** 10.2196/53108

**Published:** 2024-08-07

**Authors:** Leena R Baghdadi, Arwa A Mobeirek, Dania R Alhudaithi, Fatimah A Albenmousa, Leen S Alhadlaq, Maisa S Alaql, Sarah A Alhamlan

**Affiliations:** 1 Department of Family and Community Medicine College of Medicine King Saud University Riyadh Saudi Arabia; 2 College of Medicine King Saud University Riyadh Saudi Arabia

**Keywords:** artificial intelligence, diagnostic radiology, patients, attitudes, questionnaire, patient, attitude, diagnostic tool, diagnostic tools, AI, artificial intelligence, radiologists, prognosis, treatment, Saudi Arabia, sociodemographic factors, sociodemographic factor, sociodemographic, cross-sectional study, participant, men, women, analysis, distrust, trust

## Abstract

**Background:**

Artificial intelligence (AI) is widely used in various medical fields, including diagnostic radiology as a tool for greater efficiency, precision, and accuracy. The integration of AI as a radiological diagnostic tool has the potential to mitigate delays in diagnosis, which could, in turn, impact patients’ prognosis and treatment outcomes. The literature shows conflicting results regarding patients’ attitudes to AI as a diagnostic tool. To the best of our knowledge, no similar study has been conducted in Saudi Arabia.

**Objective:**

The objectives of this study are to examine patients’ attitudes toward the use of AI as a tool in diagnostic radiology at King Khalid University Hospital, Saudi Arabia. Additionally, we sought to explore potential associations between patients’ attitudes and various sociodemographic factors.

**Methods:**

This descriptive-analytical cross-sectional study was conducted in a tertiary care hospital. Data were collected from patients scheduled for radiological imaging through a validated self-administered questionnaire. The main outcome was to measure patients’ attitudes to the use of AI in radiology by calculating mean scores of 5 factors, distrust and accountability (factor 1), procedural knowledge (factor 2), personal interaction and communication (factor 3), efficiency (factor 4), and methods of providing information to patients (factor 5). Data were analyzed using the student *t* test, one-way analysis of variance followed by post hoc and multivariable analysis.

**Results:**

A total of 382 participants (n=273, 71.5% women and n=109, 28.5% men) completed the surveys and were included in the analysis. The mean age of the respondents was 39.51 (SD 13.26) years. Participants favored physicians over AI for procedural knowledge, personal interaction, and being informed. However, the participants demonstrated a neutral attitude for distrust and accountability and for efficiency. Marital status was found to be associated with distrust and accountability, procedural knowledge, and personal interaction. Associations were also found between self-reported health status and being informed and between the field of specialization and distrust and accountability.

**Conclusions:**

Patients were keen to understand the work of AI in radiology but favored personal interaction with a radiologist. Patients were impartial toward AI replacing radiologists and the efficiency of AI, which should be a consideration in future policy development and integration. Future research involving multicenter studies in different regions of Saudi Arabia is required.

## Introduction

### Introduction to Artificial Intelligence

Artificial intelligence (AI) is a branch of computer science focused on creating systems that mimic human intelligence, enabling machines to learn, solve problems, and understand language and visuals. The aim is to develop machines capable of performing cognitive tasks traditionally associated with human intelligence. It is anticipated that this revolution in data science and technology will provide practical advantages in many areas [1]. AI has many subsets, including algorithmic machine learning and autonomous decision-making. AI is used in diverse specialties, including health care [2]. In health care, AI decreases the workload of health care providers [3] and improves disease prevention, diagnosis, management, and treatment; thereby, improving patient outcomes and decreasing the economic burden [1,2].

Numerous studies have explored the implementation of AI in diverse aspects of medicine [4-6]. AI-based technologies have been used in surgery, observation of pulmonary metastasis on computed tomography scans, diagnosis and evaluation of diabetic retinopathy, Alzheimer disease, heart arrhythmias, onychomycosis, vertebral compressions, cerebral aneurysms, brain neoplasms, assessment of psoriasis, detection of subarachnoid hemorrhage, and for glioblastoma prognosis after bevacizumab treatment [5-8]. AI is also expected to show advancements in differentiating lung diseases and improvements in breast and skin cancer detection and screening [4,7,9]. However, to integrate AI appropriately, it is crucial to grasp the viewpoints of various stakeholders, including patients and health care professionals like students, physicians, and caregivers, regarding the use of AI in clinical practices.

### Implementation of AI in Radiology

Radiology is an important digital data generator. Due to advancements in technology, increasing rates of diagnostic procedures among older patients in an aging population, and improvements in screening programs [1,2], there is a notable rise in the number of scans to be evaluated and diagnosed. However, this demand coincides with a lack of trained radiologists in certain specialties [10]. As a result, radiologists face an increased workload, leading to marked delays in diagnosis and reduced interpretation power. Such delays could affect patients’ prognosis and treatment outcomes [10-12].

Saudi Arabia has prioritized changing this practice to be more time efficient and improve the health care system, especially for clinical cases where time is crucial for quick diagnosis and immediate management (stroke, trauma, and cancer). In radiology, a surge of evidence has demonstrated the potential of AI to outperform physicians in some clinical aspects. The use of AI would decrease the time between patient diagnosis and administration of treatment. Fewer delays in diagnosis and treatment can help mitigate legal implications, such as malpractice cases, where timely and accurate diagnoses supported by AI can minimize the risk of misdiagnoses or delayed treatments that may result in patient harm [10]. Consequently, the implementation of AI in radiology became a heated debate and a subject of discussion in around 800 related published papers in 2017 [13]. AI developers have increased efforts to provide trustworthy technologies that aid image recognition and diagnostic tasks minimizing radiologists’ workloads [12]. AI can achieve faster-customized diagnoses and recommendations equal to or superior to highly qualified radiologists for performance [4,7,11,14,15], precision, and accuracy [8], thus increasing diagnostic reliability [3].

The use of AI also involves ethical, legal, and societal concerns, which are essential and must be addressed. These involve protecting autonomy, ensuring transparency and accountability, and promoting human well-being, safety, and equity. Legal concerns include adhering to protection laws, establishing principles for the use of AI in health, and complying with bioethics regulations [16]. Recognizing these concerns, it is crucial for developers to engage all stakeholders, especially the patients that AI intends to serve [11,14,15]. The World Health Organization [16] states the public should be informed about the development of AI in health care as it will facilitate their understanding of data use and allocation and help them voice their concerns and anticipation of the use of AI technologies. The public should be encouraged to have further knowledge of AI technologies to determine those acceptable for use. Therefore, there is an increasing need to explore patient attitudes toward using AI in radiology and address social and ethical considerations.

Patients are important stakeholders in the decision-making process and understanding their perspective is necessary to ensure widespread implementation of these technological advances. By studying patients’ attitudes, we can uncover potential barriers and facilitators to the integration of AI, ultimately informing strategies to enhance patient engagement, trust, and satisfaction. Moreover, exploring patients’ viewpoints adds a crucial dimension to the discourse on AI in health care, fostering patient-centered approaches and ensuring that AI implementations align with patients’ needs and preferences [4,7]. Positive patient attitudes to AI as a diagnostic tool will indicate readiness and support, whereas a lack thereof will indicate patient education is necessary. To the best of our knowledge, no studies have examined patients’ attitudes toward the use of AI in radiology or health care in Saudi Arabia.

This study aimed to investigate the attitudes of patients toward the use of AI in diagnostic radiology at King Khalid University Hospital (KKUH) in Riyadh, Saudi Arabia, and to evaluate potential associations between patients’ attitudes and specific sociodemographic factors. Our study could influence future policy making on the integration of AI into health care by defining critical points of supervision, it will guide program development and primary education on a national level, ensuring AI implementation caters to societal needs.

## Methods

### Recruitment

This was an analytical cross-sectional study conducted between July and December 2022 at the tertiary care hospital KKUH, Riyadh, Saudi Arabia. Eligible participants were outpatients scheduled for any type of radiological imaging involving any part of the body, aged ≥18 years old, literate, and able to speak Arabic or English. Mental health patients were excluded. We used convenience sampling techniques for ease of recruitment and cost-effectiveness. Participants were approached for enrollment in the radiology waiting rooms by 1 of 6 investigators. Participants who voluntarily agreed to participate and provided their explicit consent were provided a brief description of the role of AI in diagnostic radiology and asked to complete a self-administered electronic questionnaire via Google form in Arabic or English based on their preferences. Questionnaires were completed by participants from August 9, 2022, to September 1, 2022.

### Study Variables

The main outcome of the study was patients’ attitudes to using AI in radiology at KKUH. Demographic and socioeconomic data (age, sex, nationality, marital status, education level, place of residence, family income, employment status, specialty, self-reported health status, and self-reported prior knowledge about AI, and experience of diagnostic errors) were collected.

### Survey Tools

The questionnaire had 2 sections, sociodemographic data and attitude toward AI in radiology. The first section had participant characteristics, including age, sex, nationality, marital status, education level, place of residence, family income, employment status, and field of specialization, and additional information such as self-reported health status, self-reported prior knowledge about AI, and experience of diagnostic errors.

The second section measured attitudes toward AI in radiology using a validated questionnaire (Multimedia Appendix 1) developed by Ongena et al [17]. It is a 39-item tool that calculates the average score of 5 dimensions, which are distrust and accountability (15 items), procedural knowledge (8 items), personal interaction (7 items) and communication, efficiency (5 items), and ways patients are informed of their imaging results and prognosis (4 items). Respondents assessed each item on a 5-point Likert scale ranging from “Strongly Disagree” to “Strongly Agree.” The mean of statements within each factor was then calculated. Subsequently, the scores for the 5 factors were categorized into levels of attitude: “Strongly Negative Attitude” (1.00-1.80), “Moderately Negative Attitude” (1.81-2.60), “Neutral Attitude” (2.61-3.40), “Moderately Positive Attitude” (3.41-4.20), and “Strongly Positive Attitude” (4.21-5.00), using criteria corresponding to the Likert scale values. The higher scores indicate being more negative toward the use of AI in radiology.

The tool was originally developed in English and conceptually translated into Arabic by a certified translator whose first language is Arabic and who is fluent in English. A panel of 12 experts (radiologists, public health consultants, epidemiologists, and family and community medicine consultants) reviewed the Arabic version of the questionnaire and compared it with the original English version. The panel provided several suggestions for improving the translation. A pilot study was conducted and responses from the pilot study were excluded from the analysis.

### Statistical Analysis

Based on previous reports of a 1.10 difference between participants’ attitudes toward AI [17], the minimum sample size consisted of 384 participants in total and had more than 80% power (α=.05) to detect small differences between participants. Ten percent was added to account for nonresponses, yielding a total sample size of 423 participants.

Data were analyzed using the SPSS (version 27; IBM Corp). Descriptive statistics (frequencies, percentages, means, and SDs) were used to describe the categorical and quantitative variables. The hypothesis testing was 1-tailed. Associations were determined between the patients’ attitudes to using AI in radiology and demographic and socioeconomic data (such as age, sex, nationality, marital status, and education level) through a bivariate analysis using the student *t* test for independent samples or a one-way analysis of variance followed by post hoc analysis to test for quantitative outcome variables to compare the mean values in relation to the categorical study variables, which have 2 and >2 options, respectively. Common confounders, such as sociodemographic factors, were analyzed either via student *t* test or ANOVA as appropriate. The Pearson correlation coefficient was used to determine the association between continuous quantitative study variables (age) and the outcome variables.

### Multivariable Analysis

Multivariable regression models examined whether any associations between the sociodemographic variables and common confounders and quantitative outcome variables were retained after controlling for selected covariates. Sociodemographic variables that were found statistically significant (or approaching significance level) in bivariate analysis were entered as predictors in the multivariable regression model. Four regression models were built (factors 1, 2, and 5, overall score). Factors 3 and 4 only had one significant (or approaching significance) predictor, therefore multivariable analysis was not warranted. Categorical predictors were included with the most suitable category being chosen as the reference category. For each regression model, we reported the overall model fit, significance of each predictor (omnibus ANOVA), regression coefficients (including 95% CI and standardized estimate), and checked for multicollinearity assumption (variance inflation factor values). Variance inflation factor values <5 indicate no presence of multicollinearity.

A level of significance of 0.05 was used for all inferential analyses. The 95% CI was reported where applicable. *P* values were reported for inferential tests, with *P*<.05 interpreted as statistically significant. Missing data such as occupation and social status were collected from patients’ files through the electronic system for integrated health information (e-SIHI). Invalid entries were excluded from the analysis, such as patients who had nondiagnostic radiological imaging or those who were not waiting for a radiology appointment regarding their own health.

### Ethical Considerations

The study was approved by the Institutional Review Board of the College of Medicine at King Saud University on July 24, 2022 (research project number E-22-6966). All participants were verbally informed and were given sufficient time to read the consent form and give written consent before enrollment (Multimedia Appendix 1). Participation was voluntary, and subjects retained the autonomy to withdraw from the study at any point without incurring any adverse consequences.

## Results

### Descriptive Statistics

There were 382 completed surveys with a nonresponse rate of ≈11%. Participants were 18-86 years old (mean 39.51, SD 13.26 years), 273 (71.5%) women and 109 (28.5%) men, 281 (73.6%) were married, and 293 (76.7%) were college-educated individuals. There were 180 (47.1%) employed participants and 131 (34.3%) of participants had a monthly household income of US $ 2666-5331. The participants’ detailed demographic information is in Table 1.

The participants were scheduled for ultrasound imaging (209/382, 54.7%), magnetic resonance imaging (46/382, 12%), x-rays (43/382, 11.3%), computed tomography scans (40/382, 10.5%), mammograms (20/382, 5.2%), echocardiography (18/382, 4.7%), and angiography (6/382, 1.6%). Some participants (8.6%) had previously experienced diagnostic medical errors (Table S1, Multimedia Appendix 2). Most respondents evaluated their prior knowledge of AI as average (36.1%) or very good (27.7%; Table S2, Multimedia Appendix 2). The top 3 sources of their AI information were internet sources (80.4%), social media (66.2%), and friends and peers (42.7%; Table S3, Multimedia Appendix 2).

Table 2 presents the average scores for patients’ attitudes toward AI in radiology per statement and factor. Participants were neutral in their trust of AI taking over radiologists’ diagnostic interpretation tasks for accuracy, communication, and confidentiality, 3.16 was the average score for factor 1 (distrust and accountability). The average score for factor 2 (procedural knowledge) was 4.08, signifying that patients are interested in understanding how radiological images are obtained, interpreted, and disseminated. The average score for factor 3 (personal interaction) was 4.06, indicating that patients favored personal interaction with a radiologist over AI-based communication. The average score for factor 4 (efficiency) was 2.89, suggesting patients were ambiguous about AI improving the diagnostic procedure. Factor 5 (being informed) had an average score of 3.65, showing patients favor obtaining full disclosure of their medical findings and predictions of any future diseases they might develop, in addition to full-body scans performed by AI rather than scans of selected parts of the body.

**Table 1:** Sociodemographic and health characteristics of the participants (N=382).

Characteristics		Values, n (%)
**Age group (years)**		
	18-19	8 (2.1)
	20-29	78 (20.4)
	30-39	143 (37.4)
	40-49	68 (17.8)
	≥50	85 (22.3)
**Sex**		
	Male	109 (28.5)
	Female	273 (71.5)
**Nationality**		
	Saudi	355 (92.9)
	Non-Saudi	27 (7.1)
**Residence**		
	Riyadh region	342 (89.5)
	Outside Riyadh region	40 (10.5)
**Education**		
	No formal education	3 (0.8)
	Literacy school	3 (0.8)
	Elementary education	10 (2.6)
	Intermediate education	13 (3.4)
	Secondary education	60 (15.7)
	College (Diploma, Bachelor, Master, or PhD)	293 (76.7)
**Field of specialization (n=376)**		
	Health sciences	49 (12.8)
	Scientific field	41 (10.7)
	Humanities	139 (36.4)
	Technology and computer science	26 (6.8)
	Administrative field	73 (19.1)
	Other	3 (0.8)
	None	45 (11.8)
**Employment status**		
	Student	41 (10.7)
	Employed	180 (47.1)
	Unemployed	39 (10.2)
	Retired	41 (10.7)
	Housewife	81 (21.2)
**Marital status**		
	Married	281 (73.6)
	Unmarried	73 (19.1)
	Divorced or widowed	28 (7.3)
**Monthly household income (** **US $** **)**		
	≤1333	84 (22)
	1333-2665	121 (31.7)
	2666-5331	131 (34.3)
	5332-7997	26 (6.8)
	>7997	20 (5.2)
**Self-reported health status**		
	Excellent	122 (31.9)
	Very good	159 (41.6)
	Average	89 (23.3)
	Fair	10 (2.6)
	Poor	2 (0.5)

**Table 2:** Results of the 39 questionnaire statements that provide scores for 5 factors.

Items			Score, mean^a^ (SD)
**Factor 1 (distrust and accountability)**			**3.16 (0.59)**
	1. A computer can never compete against the experience of a specialized doctor (radiologist)	3.19 (1.04)	
	2. Through human experience, a radiologist can detect more than the computer	3.30 (0.99)	
	3. Humans have a better overview than computers on what happens in my body	3.52 (0.97)	
	4. It worries me when computers analyze scans without interference of humans	3.31 (1.12)	
	5. I wonder how it is possible that a computer can give me the results of a scan	2.88 (1.11)	
	6. Artificial intelligence makes doctors lazy	2.83 (1.18)	
	7. Humans and artificial intelligence can complement each other	2.70 (1.04)	
	8. I think replacement of doctors by artificial intelligence will happen in the far future	2.98 (1.12)	
	9. I would never blindly trust a computer	3.30 (1.08)	
	10. Artificial intelligence can only be implemented to check human judgment	3.56 (1.10)	
	11. I find it worrisome that a computer does not take feelings into account	3.50 (1.13)	
	12. It is unclear to me how computers will be used in evaluating scans	3.19 (1.02)	
	13. Even if computers are better in evaluating scans, I still prefer a doctor	3.31 (1.15)	
	14. When artificial intelligence is used, my personal data may fall into the wrong hands	3.01 (1.16)	
	15. Artificial intelligence may prevent errors^b^	2.77 (0.98)	
**Factor 2 (procedural knowledge)**			**4.08 (0.71)**
	1. I find it important to have a good understanding of the results of a scan	4.16 (0.83)	
	2. I find it important to be able to ask questions personally about the results of a scan	4.17 (0.84)	
	3. I find it important to talk with someone about the results of a scan	4.19 (0.87)	
	4. I find it important that a scan provides as much information about my body as possible	4.18 (0.88)	
	5. I find it important to get the results of a scan as fast as possible	4.13 (0.85)	
	6. I find it important to ask questions on the reliability of the results	4.11 (0.92)	
	7. I find it important to be well informed about how a scan is made	4.02 (0.94)	
	8. I find it important to read how radiologists work before I get a scan	3.68 (0.99)	
**Factor 3 (personal interaction)**			**4.06** (**0.71)**
	1. When discussing the results of a scan, humans are indispensable	4.16 (0.91)	
	2. Getting the results involves personal contact	3.96 (0.93)	
	3. As a patient, I want to be treated as a person, not as a number	4.14 (0.87)	
	4. When a computer gives the result, I would miss the explanation	3.69 (1.12)	
	5. I find it important to ask questions when getting the result	4.18 (0.85)	
	6. Even when computers are used to evaluate scans, humans always remain responsible	4.13 (0.92)	
	7. Humans and artificial intelligence can complement each other	4.17 (0.94)	
**Factor 4 (efficiency)**			**2.89** (**0.46)**
	1. As far as I am concerned, artificial intelligence can replace doctors in evaluating scans^b^	3.08 (1.04)	
	2. The sooner I get the results, even when this is from a computer, the more I am at ease	3.50 (1.01)	
	3. Because of the use of artificial intelligence, fewer doctors and radiologists are required^b^	2.61 (1.07)	
	4. Evaluating scans with artificial intelligence will reduce health care waiting times^b^	2.30 (0.95)	
	5. In my opinion, humans make more errors than computers^b^	2.95 (0.93)	
**Factor 5 (being informed)**			**3.65** (**0.62)**
	1. If it does not matter in costs, a computer should always make a full-body scan instead of looking at specific body parts	3.56 (1.07)	
	2. If a computer would give the results, I would not feel emotional support	3.53 (0.98)	
	3. A computer should only look at body parts that were selected by my doctor	3.48 (1.05)	
	4. When a computer can predict that I will get a disease in the future, I want to know that no matter what	4.02 (0.97)	

^a^The mean score of statements measured on a 5-point scale (strongly disagree-strongly agree). For all factors, higher scores indicate being more negative toward the use of artificial intelligence in radiology.

^b^Items marked are recoded to measure in the same direction.

### Associations

The associations of factors with participant characteristics are presented in Table 3. Factor 1 was significantly associated with the participants’ study specialization, the level of distrust in AI was lower among individuals in the administrative field (2.88, SD 0.62) compared with those specializing in humanities (3.20, SD 0.58; *P*=.003), health sciences (3.28, SD 0.63; *P*=.005), and those with no specialty (3.36, SD 0.61; *P*<.001). Factor 1 was significantly related to employment status (*F*_4,377_=2.74, *P*=.03) and self-reported health status (*F*_3,378_=2.88, *P*=.04). However, post hoc analysis did not reveal a significant difference between employment status and the self-reported health status subgroups.

With regard to self-reported health status, a statistically significant difference was noted in the mean scores of factor 5. Participants who reported an excellent health status (3.50, SD 0.62) had significantly lower scores for factor 5 than those who evaluated their health status as average (3.74, SD 0.70, *P*=.03) or fair/poor (4.17, SD 0.59; *P*=.002).

Factors 1, 2, and 3 showed a statistically significant association with marital status on univariate analysis; divorced and widowed individuals showed a higher level of distrust of AI in radiology (factor 1, 3.46, SD 0.72), a higher need for active engagement (factor 2, 4.42, SD 0.50), and a higher appreciation for personal interaction (factor 3, 4.40, SD 0.55) when compared with married individuals (factor 1, 3.15, SD 0.57, *P*=.02; factor 2, 4.04, SD 0.75, *P=*.02; factor 3, 4.02, SD 0.74, *P*=.02). Divorced or widowed individuals had a higher level of distrust of AI in radiology than unmarried participants (factor 1, 3.08, SD 0.59, *P*=.01).

**Figure 1:**
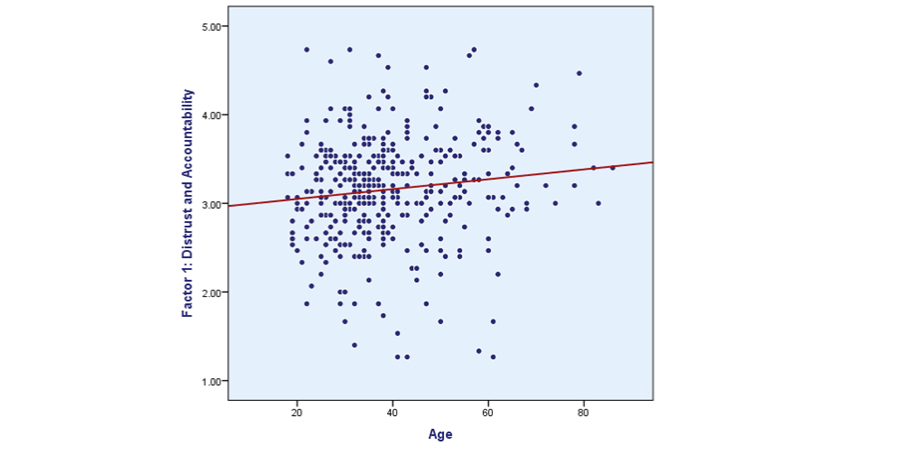
Pearson correlation between the ages of participants and factor 1 (distrust and accountability).

On univariate analysis, factor 1 was weakly positively associated with age (*r*=0.124, *P*=.02; Figure 1). When participants were categorized into different age groups, no significant differences were observed. None of the factors showed statistically significant associations with sex, nationality, residence, education level, income, or self-reported knowledge about AI.

**Table 3:** Comparison of mean scores of the 5 factors in relation to sociodemographic and health characteristics of the participants.

Variable		Factor 1	Factor 2	Factor 3	Factor 4	Factor 5
**Age group (years), mean (SD)**						
	18-19	3.09 (0.56)	4.05 (0.45)	4.29 (0.58)	2.83 (0.55)	3.72 (0.45)
	20-29	2.98 (0.39)	4.14 (0.74)	4.11 (0.62)	2.82 (0.45)	3.66 (0.64)
	30-39	3.16 (0.56)	4.11 (0.71)	4.06 (0.72)	2.90 (0.49)	3.60 (0.61)
	40-49	3.14 (0.65)	4.01 (0.66)	4.00 (0.77)	2.91 (0.46)	3.66 (0.62)
	≥50	3.25 (0.65)	4.04 (0.77)	4.05 (0.73)	2.90 (0.40)	3.71 (0.66)
*F* test (age group; 4, 377)		0.966	0.485	0.402	0.515	0.508
*P* value (age group)		.43	.75	.81	.73	.73
**Marital status, mean (SD)**						
	Unmarried	3.08 (0.59)^b^	4.13 (0.60)	4.07 (0.58)	2.88 (0.46)	3.72 (0.56)
	Married	3.15 (0.57)^b^	4.04 (0.75)^b^	4.02 (0.74)^b^	2.89 (0.46)	3.61 (0.63)
	Divorced or widowed	3.46 (0.72)^b^	4.42 (0.50)^b^	4.40 (0.55)^b^	2.88 (0.46)	3.86 (0.71)
*F* test (marital status; 2, 379)		4.449	3.918	3.592	0.004	2.514
*P* value (marital status)		.01^a^	.02^a^	.03^a^	.99	.08
**Education, mean (SD)**						
	No formal education	3.24 (0.71)	4.29 (0.40)	4.71 (0.25)	3.00 (0.40)	4.25 (0.66)
	Literacy school	3.87 (0.71)	4.54 (0.29)	4.33 (0.50)	2.80 (0.20)	4.25 (0.90)
	Elementary education	3.45 (0.79)	3.88 (1.28)	3.89 (1.14)	2.92 (0.51)	3.83 (0.69)
	Intermediate education	3.24 (0.77)	3.85 (0.83)	3.91 (0.92)	2.85 (0.52)	3.71 (0.92)
	Secondary education	3.16 (0.62)	3.96 (0.82)	4.00 (0.85)	3.03 (0.49)	3.55 (0.72)
	College	3.14 (0.57)	4.12 (0.66)	4.08 (0.65)	2.86 (0.45)	3.65 (0.58)
*F* test (education; 5, 376)		1.482	1.270	0.973	1.516	1.643
*P* value (education)		.20	.28	.43	.18	.15
**Field of specialization, mean (SD)**						
	Humanities	3.20 (0.58)^b^	4.10 (0.74)	4.07 (0.69)	2.88 (0.46)	3.60 (0.62)
	Health sciences	3.28 (0.63)^b^	4.19 (0.65)	4.12 (0.59)	2.86 (0.51)	3.68 (0.57)
	Scientific field	3.06 (0.47)	4.18 (0.57)	4.19 (0.62)	2.86 (0.42)	3.79 (0.57)
	Technology and computer science	3.21 (0.44)	4.14 (0.58)	4.14 (0.70)	2.66 (0.41)	3.78 (0.59)
	Administrative	2.88 (0.62)^b^	4.03 (0.71)	3.96 (0.75)	2.99 (0.44)	3.57 (0.59)
	Other	3.51 (0.17)	4.33 (0.58)	4.52 (0.50)	2.80 (0.35)	4.00 (0.50)
	None	3.36 (0.61)^c^	3.92 (0.89)	3.94 (0.87)	2.92 (0.47)	3.70 (0.81)
*F* test (field of specialization; 6, 369)		4.563	0.865	1.006	1.730	1.077
*P* value (field of specialization)		<.001^a^	.52	.42	.11	.38
**Employment status, mean (SD)**						
	Student	3.01 (0.51)	4.01 (0.71)	3.97 (0.64)	2.96 (0.51)	3.65 (0.68)
	Employed	3.12 (0.60)	4.12 (0.68)	4.06 (0.73)	2.88 (0.50)	3.63 (0.63)
	Unemployed	3.07 (0.61)	4.26 (0.60)	4.18 (0.59)	2.86 (0.41)	3.72 (0.49)
	Retired	3.31 (0.53)	4.02 (0.68)	4.05 (0.64)	2.92 (0.34)	3.73 (0.53)
	Housewife	3.29 (0.62)	3.97 (0.85)	4.06 (0.77)	2.87 (0.43)	3.61 (0.68)
*F* test (employment status; 4, 377)		2.740	1.463	0.432	0.365	0.408
*P* value (employment status)		.03^a^	.21	.79	.83	.80
**Self-reported health status, mean (SD)**						
	Excellent	3.05 (0.52)	4.03 (0.82)	3.96 (0.84)	2.96 (0.49)	3.50 (0.62)^b^
	Very good	3.20 (0.57)	4.11 (0.61)	4.10 (0.61)	2.84 (0.43)	3.67 (0.55)
	Average	3.20 (0.65)	4.06 (0.74)	4.09 (0.67)	2.87 (0.48)	3.74 (0.70)^b^
	Fair/poor^d^	3.46 (0.90)	4.36 (0.68)	4.32 (0.73)	2.88 (0.34)	4.17 (0.59)^b^
*F* test (self-reported health status; 3, 378)		2.878	0.948	1.520	1.517	6.042
*P* value (self-reported health status)		.04^a^	.42	.21	.21	<.001^a^
**Self-reported AI knowledge, mean (SD)**						
	Excellent	3.13 (0.66)	4.16 (0.82)	4.10 (0.82)	2.90 (0.55)	3.66 (0.74)
	Very good	3.09 (0.52)	4.07 (0.64)	4.06 (0.66)	2.85 (0.48)	3.60 (0.58)
	Average	3.18 (0.60)	4.10 (0.68)	4.09 (0.69)	2.89 (0.47)	3.64 (0.63)
	Fair	3.20 (0.56)	3.99 (0.79)	3.95 (0.72)	2.92 (0.35)	3.64 (0.61)
	Poor	3.24 (0.73)	4.08 (0.81)	4.07 (0.75)	2.89 (0.36)	3.83 (0.56)
*F* test (self-reported AI knowledge; 4, 377)		0.714	0.422	0.456	0.240	0.868
*P* value (eslf-reported AI knowledge)		.58	.79	.77	.92	.48
**Monthly household income US$, mean (SD)**						
	≤1,333	3.21 (0.59)	4.03 (0.80)	3.97 (0.78)	2.93 (0.45)	3.64 (0.76)
	1,333-2,665	3.12 (0.60)	4.02 (0.72)	4.02 (0.68)	2.93 (0.46)	3.60 (0.58)
	2,666-5,331	3.16 (0.61)	4.11 (0.69)	4.10 (0.73)	2.84 (0.43)	3.66 (0.61)
	5,332-7,997	3.12 (0.59)	4.23 (0.63)	4.25 (0.59)	2.74 (0.55)	3.71 (0.51)
	>7,997	3.19 (0.51)	4.24 (0.48)	4.17 (0.56)	2.90 (0.57)	3.80 (0.48)
*F* test (monthly household income; 6, 369)		0.301	0.883	1.119	1.500	0.571
*P* value (monthly household income)		.88	.47	.35	.20	.68
**Sex, mean (SD)**						
	Male	3.09 (0.59)	4.00 (0.76)	3.99 (0.74)	2.90 (0.48)	3.65 (0.63)
	Female	3.18 (0.60)	4.11 (0.70)	4.09 (0.69)	2.88 (0.45)	3.65 (0.62)
*t* value (sex; 380)		–1.310	1.463	0.432	0.365	0.408
*P* value (sex)		.19	.21	.79	.83	.80
95% CI (sex)		–0.220 to 0.044	–0.271 to 0.047	–0.261 to 0.054	–0.089 to 0.116	–0.142 to 0.136
**Nationality, mean (SD)**						
	Saudi	3.14 (0.59)	4.07 (0.72)	4.06 (0.72)	2.89 (0.47)	3.64 (0.63)
	Non-Saudi	3.33 (0.68)	4.23 (0.62)	4.11 (0.54)	2.86 (0.38)	3.71 (0.57)
*t* value (nationality; 380)		–1.528	–1.099	–0.341	0.312	–0.551
*P* value (nationality)		.13	.27	.73	.76	.58
95% CI (nationality)		–0.414 to 0.052	–0.437 to 0.124	–0.326 to 0.230	–0.152 to 0.209	–0.314 to 0.176
**Residence, mean (SD)**						
	Riyadh region	3.16 (0.57)	4.10 (0.68)	4.06 (0.70)	2.89 (0.45)	3.65 (0.61)
	Outside Riyadh region	3.15 (0.79)	3.89 (0.93)	4.11 (0.79)	2.83 (0.54)	3.62 (0.75)
*t* value (residence; 380)		0.096	1.816	–0.436	0.812	0.326
*P* value (residence)		.92	.07	.66	.42	.74
95% CI (residence)		–0.183 to 0.208	–0.018 to 0.450	–0.284 to 0.181	–0.089 to 0.213	–0.171 to 0.239

^a^A significant difference between means within a variable.

^b^Groups with a significant mean difference at the .05 level.

^c^Groups with a significant mean difference at the 0.001 level.

^d^“fair” and “poor” categories were combined for analysis purposes.

### Regression Models

Overall, model 1 (factor 1, distrust and accountability) was statistically significant (*F*_15,356_=3.48, *P*<.001) with a coefficient of determination (*R*^2^) of 0.128. The model explains a 12.8% variability in factor 1 scores. Out of 5 predictors (age, marital status, field of specialization, employment, and self-reported health status), only marital status and field of specialization were statistically significant (*P*=.04 and *P*<.001, respectively). Controlling for other factors in the model, participants who specialized in scientific and administrative fields still showed lower levels of distrust compared with those with no specialty; the scores were 0.27 lower (*P*=.047) and 0.44 lower (*P*<.001) than that of no-specialty participants, respectively. Divorced or widowed participants also showed higher scores when compared with married participants by 0.29 (*P*=.01), controlling for other factors in the model (Table 4).

Overall, model 2 (factor 2, procedural knowledge) was statistically significant (*F*_3,378_=3.41, *P*=.02) with a coefficient of determination (*R*^2^) of 0.026. The model explains only 2.6% variability in factor 2 scores. Out of 2 predictors (marital status and living region), only marital status was statistically significant (*P*=.03). Divorced or widowed participants showed factor 2 scores 0.36 higher compared with married participants (*P*=.01), controlling for living region (Table 4).

Overall, model 3 (factor 5, being informed) was statistically significant (*F*_6,374_=3.99, *P*<.001) with a coefficient of determination (*R*^2^) of 0.060. The model explains only 6.0% variability in factor 5 scores. Out of 3 predictors (age, marital status, and self-reported health status), only self-reported health status was statistically significant (*P*=.004).

Compared with excellent health status, participants with very good, average, fair/poor health status reported significantly higher factor 5 scores by 0.17 (*P*=.02), 0.23 (*P=*.01), and 0.58 (*P=*.002) respectively, controlling for age and marital status (Table 4).

Overall, model 4 (overall score) was statistically significant (*F*_10,362_=3.22, *P*<.001) with a coefficient of determination (*R*^2^) of 0.082. The model explains 8.2% variability in the overall scores. Out of 3 predictors (marital status, field of specialization, and self-reported health status, only marital status and health status remained statistically significant (*P*=.004 and *P*=.02 respectively). Divorced or widowed participants reported overall scores 0.28 higher compared with married participants (*P*<.001), controlling for field of specialization and self-reported health status. Compared with excellent health status, participants with very good, average, fair/poor health status reported significantly higher overall scores by 0.13 (*P*=.01), 0.12 (*P*=.04), and 0.29 (*P*=.02) respectively, controlling for field of specialization and marital status (Table 4).

**Table 4:** Multivariable models of mean scores of factors 1, 2, and 5 and overall score.

				Model 1 (DV^a^: Factor 1 score [Distrust and accountability])	Model 2 (DV: Factor 2 score [Procedural knowledge])		Model 3 (DV: Factor 5 score [Being informed])		Model 4 (DV: Overall score)
**Model fit**									
	*F* test (*df*)		3.48 (15, 356)		3.41 (3, 378)	3.99 (6, 374)		3.22 (10, 362)	
	*P* value		<.001		.02	<.001		<.001	
	*R* ^2^		0.128		—^b^	0.060		0.082	
**Age (years)**									
	RC^c^ (95% CI)		0.001 (–0.01 to 0.01)		—	0.003 (–0.002 to 0.01)		—	
	*P* value		.87		—	.27		—	
	*R* ^2^		—		0.026	—		—	
**Marital status (ref^d^: married)**									
	**RC (** **95% CI** **)**								
		Unmarried	0.05 (–0.13 to 0.23)		0.08 (–0.10 to 0.27)	0.16 (–0.02 to 0.34)		0.02 (–0.09 to 0.13)	
		Divorced/widowed	0.29^e^ (0.06 to 0.53)		0.36^e^ (0.08 to 0.64)	0.19 (–0.05 to 0.43)		0.28^f^ (0.11 to 0.45)	
	*P* value		.04		.03	.08		.004	
**Specialty (ref: none)**									
	**RC (** **95% CI** **)**								
		Humanities	–0.12 (–0.33 to 0.09)		—	—		0.02 (–0.12 to 0.16)	
		Health sciences	0.03 (–0.23 to 0.30)		—	—		0.10 (–0.07 to 0.27)	
		Scientific field	–0.27^e^ (–0.52 to –0.004)		—	—		0.02 (–0.16 to 0.19)	
		Technology and Computer science	–0.03 (–0.33 to 0.26)		—	—		0.06 (–0.14 to 0.26)	
		Administrative field	–0.44^f^ (–0.68 to –0.20)		—	—		–0.12 (–0.28 to 0.03)	
	*P* value		<.001		—	—		—	
**Employment (ref: unemployed)**									
	**RC (** **95% CI** **)**								
		Student	–0.07 (–0.35 to 0.20)		—	—		—	
		Employed	0.11 (–0.11 to 0.32)		—	—		—	
		Retired	0.28 (–0.05 to 0.61)		—	—		—	
		Housewife	0.16 (–0.08 to 0.40)		—	—		—	
	*P* value		.28		—	—		—	
**Region (ref: outside Riyadh)**									
	**RC (** **95% CI** **)**								
		Riyadh region	—		0.18 (–0.05 to 0.42)	—		—	
	*P* value		—		.13	—		—	
**Health status (ref: excellent)**									
	**RC (** **95% CI** **)**								
		Very good	0.14 (–0.0004 to 0.28)		—	0.17^e^ (0.02 to 0.32)		0.13^e^ (0.03 to 0.23)	
		Average	0.13 (–0.04 to 0.29)		—	0.23^e^ (0.05 to 0.40)		0.12^e^ (0.01 to 0.24)	
		Fair/poor	0.28 (–0.07 to 0.64)		—	0.58^g^ (0.21 to 0.96)		0.29^e^ (0.04 to 0.53)	
	*P* value		.16		—	.004		.02	

^a^DV: dependent variable.

^b^Not applicable.

^c^RC: regression coefficient.

^d^ref: reference category.

^e^*P*<.05.

^f^*P*<.001.

^g^*P*<.01.

## Discussion

### Principal Findings

To the best of our knowledge, this is the first study in Saudi Arabia to examine the attitudes to AI as a diagnostic tool from the patient’s perspective. In this cross-sectional study with 382 participants, patients in the radiology waiting rooms at KKUH had a moderately positive attitude toward the use of AI as a diagnostic tool in radiology. This was similar to our hypothesis with reference to Jutzi et al [8] and Young et al [18] and contrary to Ongena et al [17] and Lennartz et al [4].

Regarding factor 1 (distrust and accountability), even though patients were neutral in their trust of AI taking over radiologists’ diagnostic interpretation tasks; they believe that AI might enhance the accuracy of radiological diagnosis. This finding is similar to the conclusion by Jutzi et al [8], Young et al [18], and Lennartz et al [4] that AI is perceived to enhance the accuracy of radiological diagnosis and uses the latest in diagnostic procedures. As we had hypothesized that patients would show a positive attitude toward perceiving the knowledge behind radiological diagnosis and personal interaction with average scores of around 4.5 and 4.4, respectively; and in line with previous studies [4,17,18], we found that patients were interested in understanding how radiological images are obtained, interpreted, and disseminated, they favored personal interaction with a radiologist over AI-based communication, were ambiguous about AI improving the diagnostic procedure, and favored obtaining full disclosure of their medical findings and predictions of future diseases they might develop, in addition to full-body scans performed by AI rather than scans of selected body parts.

With regards to the associations between age and patients’ attitudes toward AI as a diagnostic tool, our study showed a weak positive association between age and factor 1 (distrust and accountability) on univariate analysis although multivariable analysis showed no statistical significance. Ongena et al [17] indicated that age was weakly positively associated with factor 2 (procedural knowledge) and weakly negatively associated with factor 4 (efficiency). Young et al [18] reported that younger university students had more positive attitudes toward AI as a diagnostic tool and aimed to use AI as a diagnostic tool in radiology. Contrary to the findings of Young et al [18], who reported that males were more accepting of AI as a radiological diagnostic tool and Jutzi et al [8] who reported female participants had a more positive attitude toward the use of AI in radiology than males, our study and that of Ongena et al [17] showed no significant associations between sex and the factors determining attitudes to AI as a diagnostic tool in radiology.

While Ongena et al [17] observed an increase in trust in AI-based technologies with higher levels of education, our study found no significant association between education levels and patients’ attitudes toward AI use in radiology. Nevertheless, our results align with the findings by Jutzi et al [8]; the similarity in educational backgrounds, with a notable percentage of participants in both studies holding undergraduate or postgraduate degrees (121 participants, 40.6% in Jutzi et al [8] and 293 participants, 76% in our study), coupled with a middle-aged demographic, suggests a potential wariness towards AI. This wariness may stem from concerns about privacy invasion, or limited digital skills and financial resources required for technology use [19].

The field of specialization showed a statistically significant association (*P*<.001) with patients’ trust in the use of AI in radiology. Participants in scientific and administrative fields reported lower levels of distrust in AI compared with participants with no specialty (*P*<.001). People who are well educated in a certain subject tend to build more trust in it. Thus, AI experts showed greater confidence and positivity in their views about AI implementation in medicine and health care when compared with the general public in the United States [20]. These results might be due to their previous exposure to AI for work or study, which facilitated the intention to use it in health care.

Participants who reported excellent health status have also expressed a higher need to obtain full disclosure and be informed by the AI diagnostic tool about their overall health status when compared with participants who reported their health status as average or fair/poor (*P*<.05). This was consistent with Lennartz et al [4], who reported that patients with severe disease had a negative attitude toward the use of AI in diagnosis. However, Jutzi et al [8] observed that patients with melanomas were more likely to accept the AI diagnosis than healthy people.

Although the relationship between prior knowledge of AI and patients’ attitudes toward the use of AI in radiology is the most influential determinant of its acceptance in radiological diagnosis [4], such a relationship was not statistically significant (*P*>.05) in our study. This could be a result of our participants’ age and level of education, which made them less interested in AI and learning about it [19].

Divorced or widowed participants had a higher level of distrust of AI use in radiology and expressed a greater need for active engagement when compared with married participants. This could be due to the minimal health assistance they receive from family members in their household. Without the support of a partner, they carry a heavier burden of disease and could be unaware of the minor health-related details that are usually picked up by a partner [21]. Divorced patients are more prone to illness anxiety disorders, including hypochondriasis [22,23].

According to the World Health Organization, efficient implementation of AI requires a good interpretation of the patient’s attitudes toward the use of AI in medicine in order to build their trust. One of the objectives of this study is to understand patients’ perspectives on these technologies to ensure their widespread implementation. This research may aid in adding insight into future integration policies and ensuring the suitability of AI programs to meet societal needs. The strength of this study is that we recruited participants from a large specialized referral hospital in Riyadh. Thus, the population of this study had different health statuses and needed different scans. We also considered sociodemographic differences and social determinants of health.

### Limitations

This study is subject to certain limitations, notably selection bias introduced by the convenience sampling method and potential variability in participants’ comprehension of the topic. The selection of waiting rooms for our convenience sampling approach might be influenced by several factors, including the frequency of x-ray and magnetic resonance imaging examinations performed in each facility, weighting time as well as the availability of waiting areas conducive to survey administration. While x-ray examinations are indeed more commonly performed procedures, our higher percentage of participants waiting for magnetic resonance imaging scans (or longer waiting time) may be attributed to the specific scheduling patterns and patient volumes observed during the data collection period. Additionally, variations in appointment scheduling and patient flow within different departments or clinics may have influenced the distribution of participants across waiting areas. Future research in this field should include a multicenter study population and studies that examine the predictors of distrust among patients in different hospitals, as well as identify useful methods for addressing the lack of knowledge and misconceptions that few patients hold with regard to AI.

### Conclusions

In conclusion, patients were keen to understand the work of AI in radiology but favored personal interaction with a radiologist. If an AI system was implemented, patients would prefer full-body scans and full disclosure of medical findings. Patients were impartial toward AI replacing radiologists and the efficiency of AI. This preference expressed by patients for AI could have implications for clinicians and policymakers. Clinicians may consider incorporating these preferences into the design and implementation of AI systems in radiology, ensuring that the technology aligns with patient preferences for imaging procedures and information disclosure. Policymakers, on the other hand, may use this feedback to inform regulations and guidelines surrounding the use of AI in health care, emphasizing patient-centric approaches and ethical considerations in the integration of AI technologies. Therefore, our findings provide insight for future integration policies and help adapt AI to societal needs.

## Appendix

Multimedia Appendix 1 Questionnaire and consent - English.

Multimedia Appendix 2 Supplementary results' tables.

## Abbreviations

AI: artificial intelligence
e-SIHI: electronic system for integrated health information
KKUH: King Khalid University Hospital
