# Supplementary material for: Patients’ Attitudes Toward the Use of Artificial Intelligence as a Diagnostic Tool in Radiology in Saudi Arabia: Cross-Sectional Study
Source: JMIR Hum Factors. 2024 Aug 7;11:e53108. doi: 10.2196/53108 (PMC11339559; doi:10.2196/53108)
Supplement: Multimedia Appendix 1 [file humanfactors_v11i1e53108_app1.pdf]

Introduction

Dear participant,

We invite you to participate in our survey study titled “Patients’ Attitudes Towards the Use of Artificial Intelligence as a Diagnostic Tool in Radiology”. As developments in the field of computers go very fast, in the future, many medical procedures can be performed independently by a computer. This assistance from computers is also referred to as ‘artificial intelligence’.

Please give us less than 7 minutes of your precious time to complete this survey. Be assured that all answers you provide will be kept confidential, and used for research purposes only. Feel free to contact the principal investigator Dr. Leena Baghdadi on 0501235269 to have your questions answered. If you agree to participate in this survey, please click "Yes" to begin. This study has been approved by the Ethical Committee for Scientific Research at the College of Medicine, King Saud University with No. "E-22-6966".

Exclusion Criteria:

I agree to participate in the study:
- Yes
- No; reason: _____
- I have already filled this survey

Are you waiting for a radiology appointment that concerns your health?
- Yes
- No

Which imaging modality will be used in your screening test?
- X-ray
- Computed tomography scan
- Magnetic resonance imaging
- Ultrasound
- Mammogram
- Echocardiography
- Nuclear scan
- Angiography
- Other: _____

Have you ever experienced a diagnostic medical error?

- Yes, I have experienced a diagnostic medical error
- No

Is this your first time having radiological imaging?

- Yes, this is my first radiological imaging
- No

Are you currently admitted to the hospital (inpatient)?

- Yes
- No

What is your medical file number at King Khalid University medical hospital?:_____

A) Socio-demographic Questions

1. **Age:**

2. **Gender:**
    - Male
    - Female

3. **Nationality:**
    - Saudi
    - Other: _____

4. **Region:**
    - Al-Bahah region
    - Al-Jawf region
    - Northern Borders region
    - Riyadh region
    - Eastern region
    - Al-Qassim region
    - Medina region
    - Tabuk region
    - Jizan region
    - Ha’il region
    - Asir region
    - Mecca region
    - Najran region

5. **Region (for Riyadh residents):**
   - Not from Riyadh
   - Central region
   - Eastern region
   - Western region
   - Northern region
   - Southern region
   - Provinces outside Riyadh city (Al-Aflaj, Al-Hareeg, Al-Kharj, Al-Dir'iyyah, Al-Duwadmi, Al-Zulfy, Al-Sulayyil, Al-Ghat, Al-Gway'iyyah, Al-Majma'ah, Al-Muzahmiyyah, Thadig, Huraymila, Hotat Bani Tamim, Rimah, Shagra, Dhruma, 'Afif, Wadi Ad-Dawasir - other)

6. **Highest level of education completed:**
   - No formal education
   - Literacy school
   - Primary education (elementary school)
   - Intermediate education (middle school)
   - Secondary education (high school)
   - Diploma
   - Bachelor
   - Master
   - PhD

7. **Field of specialization (career or study):**
   - Military science
   - Health sciences
   - Scientific field
   - Technology & computer sciences
   - Engineering
   - Administrative field (business & administration)
   - Food & agriculture
   - Islamic & Qur'anic studies
   - Translation & linguistics
   - Humanities (education - literature - media - law - political sciences - social work)
   - Design & arts
   - Sports & physical education
   - Other: _________

8. **Marital status:**
    - Unmarried
    - Married
    - Divorced / Separated
    - Widowed

9. **Monthly household income (in Saudi Riyals):**
    - Less than 5,000 Riyal
    - 6,000 - 10,000 Riyal
    - 11,000 - 15,000 Riyal
    - 16,000 - 20,000 Riyal
    - 21,000 - 25,000 Riyal
    - 26,000 - 30,000 Riyal
    - 31,000 - 35,000 Riyal
    - 36,000 - 40,000 Riyal
    - 41,000 - 45,000 Riyal
    - 46,000 - 50,000 Riyal
    - More than 50,000 Riyal

10. **Current employment status:**
    - Student
    - Employed in the public sector
    - Employed in the private sector
    - Self-employed
    - Not-employed
    - Retired
    - Housewife

11. **Your current health status:**
    - Excellent
    - Very good
    - Average
    - Fair
    - Poor

12. **Your knowledge about artificial intelligence:**
    - Excellent
    - Very good
    - Average
    - Fair
    - Poor

13. Source of information about artificial intelligence: (please answer with yes or no)

- Family members (Yes/No)
- Friends and peers (Yes/No)
- Healthcare personnel (Yes/No)
- Books (Yes/No)
- Journals (Yes/No)
- Newspaper (Yes/No)
- Internets sources (Yes/No)
- Social media such as twitter, Facebook etc. (Yes/No)
- Other (Yes/No)

B) Patient Acceptance of AI in Radiology

The following questions are about radiology. A radiologist is a medical specialist who uses medical imaging tests to diagnose diseases. In the future, it is expected that artificial intelligence and computers will assess radiological scans independently. We would like to know what you think about the future use of these technologies in assessing radiological scans.

Factor 1: "distrust and accountability" - 15 items:

1. A computer can never compete against the experience of a specialized doctor (radiologist)
2. Through human experience, a radiologist can detect more than the computer
3. Humans have a better overview than computers on what happens in my body
4. It worries me when computers analyze scans without interference of humans
5. I wonder how it is possible that a computer can give me the results of a scan
6. Artificial intelligence makes doctors lazy
7. I think radiology is not ready for implementing artificial intelligence in evaluating scans
8. I think replacement of doctors by artificial intelligence will happen in the far future
9. I would never blindly trust a computer
10. Artificial intelligence can only be implemented to check human judgment
11. I find it worrisome that a computer does not take feelings into account
12. It is unclear to me how computers will be used in evaluating scans
13. Even if computers are better in evaluating scans, I still prefer a doctor
14. When artificial intelligence is used, my personal data may fall into the wrong hands
15. Artificial intelligence may prevent errors

Factor 2: “procedural knowledge” - 8 items:

1. I find it important to have a **good understanding** of the results of a scan
2. I find it important to be able to ask questions **personally** about the results of a scan
3. I find it important to **talk** with someone about the results of a scan
4. I find it important that a scan provides as **much information** about my body as possible
5. I find it important to get the results of a scan as **fast** as possible
6. I find it important to ask questions on the **reliability** of the results
7. I find it important to be **well informed** about how a scan is made
8. I find it important to **read** how radiologists work before I get a scan

Factor 3: “personal interaction” - 7 items:

1. When discussing the results of a scan, humans are indispensable
2. Getting the results involves personal contact
3. As a patient, I want to be treated as a person, not as a number
4. When a computer gives the result, I would miss the explanation
5. I find it important to ask questions when getting the result
6. Even when computers are used to evaluate scans, humans always remain responsible
7. Humans and artificial intelligence can complement each other

Factor 4: “efficiency” - 5 items:

1. As far as I am concerned, artificial intelligence can replace doctors in evaluating scans
2. The sooner I get the results, even when this is from a computer, the more I am at ease
3. Because of the use of artificial intelligence, fewer doctors and radiologists are required
4. Evaluating scans with artificial intelligence will reduce healthcare waiting times
5. In my opinion, humans make more errors than computers

Factor 5: “being informed” - 4 items:

1. If it does not matter in costs, a computer should always make a full body scan instead of looking at specific body parts
2. If a computer would give the results, I would not feel emotional support
3. A computer should only look at body parts that were selected by my doctor
4. When a computer can predict that I will get a disease in the future, I want to know that no matter what
