# Supplementary material for: Patients’ Attitudes Toward the Use of Artificial Intelligence as a Diagnostic Tool in Radiology in Saudi Arabia: Cross-Sectional Study
Source: JMIR Hum Factors. 2024 Aug 7;11:e53108. doi: 10.2196/53108 (PMC11339559; doi:10.2196/53108)
Supplement: Multimedia Appendix 2 [file humanfactors_v11i1e53108_app2.pdf]

Appendix 2: Supplementary Materials

Table S1: Previous experience with diagnostic errors

	n (%)
Yes	33 (8.6)
No	349 (91.4)

Table S2: Prior knowledge about AI

	n (%)
Excellent	50 (13.1)
Very good	106 (27.7)
Average	138 (36.1)
Fair	55 (14.4)
Poor	33 (8.6)

Table S3: Sources of information about AI

**Source**	**Yes** n (%)	**No** n (%)
Family members	137 (35.9)	245 (64.1)
Friends and peers	163 (42.7)	219 (57.3)
Healthcare personnel	97 (25.4)	285 (74.6)
Books	116 (30.4)	266 (69.6)
Journals	109 (28.5)	273 (71.5)
Newspaper	47 (12.3)	335 (87.7)
Internet sources	307 (80.4)	75 (19.6)
Social media such as Twitter, Facebook, etc.	253 (66.2)	129 (33.8)
Other	113 (29.6)	269 (70.4)
